# Expression of R345W-Fibulin-3 Induces Epithelial-Mesenchymal Transition in Retinal Pigment Epithelial Cells

**DOI:** 10.3389/fcell.2020.00469

**Published:** 2020-06-19

**Authors:** Mi Zhou, Sarah R. Weber, Yuanjun Zhao, Han Chen, Alistair J. Barber, Stephanie L. Grillo, Carson A. Wills, Hong Gang Wang, John D. Hulleman, Jeffrey M. Sundstrom

**Affiliations:** ^1^Department of Ophthalmology, Penn State Hershey College of Medicine, Hershey, PA, United States; ^2^TEM Facility, Penn State Hershey College of Medicine, Hershey, PA, United States; ^3^Division of Hematology and Oncology, Department of Pediatrics, Penn State Hershey College of Medicine, Hershey, PA, United States; ^4^Department of Ophthalmology, UT Southwestern Medical Center, Dallas, TX, United States

**Keywords:** RPE, dedifferentiation, EMT, protein misfolding, macular degeneration

## Abstract

**Purpose:**

To investigate the role of protein misfolding in retinal pigment epithelial (RPE) cell dysfunction, the effects of R345W-Fibulin-3 expression on RPE cell phenotype were studied.

**Methods:**

Primary RPE cells were cultured to confluence on Transwells and infected with lentivirus constructs to express wild-type (WT)- or R345W-Fibulin-3. Barrier function was assessed by evaluating zonula occludens-1 (ZO-1) distribution and trans-epithelial electrical resistance (TER). Polarized secretion of vascular endothelial growth factor (VEGF), was measured by Enzyme-linked immunosorbent assay (ELISA). Differentiation status was assessed by qPCR of genes known to be preferentially expressed in terminally differentiated RPE cells, and conversion to an epithelial–mesenchymal transition (EMT) phenotype was assessed by a migration assay.

**Results:**

Compared to RPE cells expressing WT-Fibulin-3, ZO-1 distribution was disrupted and TER values were significantly lower in RPE cells expressing R345W-Fibulin-3. In cells expressing mutant Fibulin-3, VEGF secretion was attenuated basally but not in the apical direction, whereas Fibulin-3 secretion was reduced in both the apical and basal directions. Retinal pigment epithelial signature genes were downregulated and multiple genes associated with EMT were upregulated in the mutant group. Migration assays revealed a faster recovery rate in ARPE-19 cells overexpressing R345W-Fibulin-3 compared to WT.

**Conclusions:**

The results suggest that expression of R345W-Fibulin-3 promotes EMT in RPE cells.

## Introduction

Retinal pigment epithelial (RPE) cells, photoreceptors, and the choroid form a functional unit required for healthy vision. The unique structure and polarity of the RPE monolayer is critical to maintain photoreceptors. Atrophy and dysfunction of the RPE with subsequent loss of photoreceptors plays a fundamental role in numerous retinal degenerations. RPE dysfunction manifests as a loss of barrier function, disrupted polarization, and downregulated expression of RPE signature genes and microRNA-204/211 (Wang et al., 2010; Adijanto et al., 2012). Recent evidence suggests that RPE cells lose terminal differentiation and acquire a mesenchymal cell phenotype in several retinal degenerations (Ghosh et al., 2018; Goldberg et al., 2018; Wu et al., 2019). Furthermore, misfolded proteins accumulate in RPE cells with age, but the relationship between misfolded protein accumulation and RPE epithelial–mesenchymal transition (EMT) remains unclear.

During EMT, cells lose their epithelial-specific markers and increase expression of mesenchymal drivers. Historically, it is thought that in healthy tissues, fully differentiated epithelial cells exert specific functions and maintain their phenotype after development. However, EMT can be activated under pathological circumstances, facilitating epithelial cells to obtain an enhanced migration ability and increase their production of extracellular matrix (ECM) components. Clinical evidence suggests that RPE cells undergo EMT with upregulated mesenchymal cell markers and enhanced migration ability in several degenerative retinal diseases, including inherited rod-cone degenerations, inherited macular degeneration, age-related macular degeneration (AMD), and proliferative vitreoretinopathy (Hirasawa et al., 2011; Tamiya and Kaplan, 2016; Ghosh et al., 2018; Goldberg et al., 2018; Wu et al., 2018). Optical coherence tomography (OCT) has detected intraretinal hyper-reflective foci (HRF) in various retinal diseases, which have been interpreted as migratory RPE cells, macrophages or hard exudates (Kuroda et al., 2014; Piri et al., 2015; Miura et al., 2017). Combined polarimetry with auto-fluorescence to discriminate between RPE cells, inflammatory cells, and hard exudates, revealed that a portion of HRF showed signs of RPE migration in patients with the early stages of AMD, substantiating the possibility that RPE cells have the capacity to migrate into the neuroretina (Miura et al., 2017).

Intracellular misfolded protein accumulation has been shown to induce loss of differentiation through activation of the unfolded protein response (UPR; Pallet, 2012; Lamouille et al., 2014). Similarly, stress due to intracellular amyloid-β aggregates has been shown to dissociate tight junctions (Park et al., 2014). These studies are consistent with data in other cells and systems that have revealed an interaction between the UPR and EMT (Zhong et al., 2011; Lenna and Trojanowska, 2012; Pang et al., 2016; Santamaria et al., 2019). The UPR is regulated by three endoplasmic reticulum (ER) transmembrane sensors, IRE1α, PERK, and ATF6 (Lin et al., 2007; Walter and Ron, 2011). The activation of IRE1α-XBP1 signaling promotes EMT in breast, lung, and liver tissues (Li et al., 2015; Mo et al., 2015; Cuevas et al., 2017; Liu et al., 2019). Several studies have shown that the UPR and TGF-β-induced EMT signaling pathways interact at the level of c-Jun N-terminal kinase and p38 mitogen-activated protein kinase (JNK/p38-MAPK) in an IRE1-dependent manner (Urano et al., 2000; Santibanez, 2006; Liu et al., 2019). To date, the potential role of the IRE1α-XBP1 in RPE cell dysfunction has not been explored.

Fibulin-3 is a secretory protein and contains six epidermal growth factor (EGF)-like domains and a fibulin domain (Zhang and Marmorstein, 2010). Fibulin-3 is expressed throughout the body including the eyes. A single arginine-to-tryptophan point mutation, R345W, is responsible for the inherited macular degeneration, Doyne honeycomb retinal dystrophy (Stone et al., 1999; DHRD; Garland et al., 2014; Fernandez-Godino et al., 2018), and is known to impair Fibulin-3 secretion from RPE (Marmorstein et al., 2002; Roybal et al., 2005; Hulleman and Kelly, 2015). Prior studies have also shown that R345W-Fibulin-3 is misfolded and migrates faster than the wild-type (WT) form on non-reducing gels (Marmorstein et al., 2002). Thus, overexpression of R345W-Fibulin-3 provides a useful model to study the impact of protein misfolding on RPE phenotype and dysfunction.

In this study, we investigated the role of fibulin-3 protein misfolding on RPE cell dedifferentiation and dysfunction. We infected primary RPE cells with lentivirus carrying luciferase-tagged wild-type or R345W-Fibulin-3. Our results show that expression of R345W-Fibulin-3 activates ER stress via the IRE1α/XBP1 pathway, which in turn attenuates RPE cell differentiation, indicated by disrupted tight junctions, impaired polarization, and downregulation of RPE signature gene expression. Moreover, we report that the expression of R345W-Fibulin-3 upregulates EMT markers and enhances the migration ability of RPE cells.

## Materials and Methods

### Plasmids

Lentiviral constructs containing naturally secreted *Gaussia* Luciferase (GLuc) and GLuc tagged wild type or R345W Fibulin-3 were described previously (Hulleman et al., 2013). ViraPower^TM^ Lentiviral Expression systems (Thermo Fisher Scientific, Waltham, MA, United States) were used to produce Lentiviruses in 293T cells by calcium phosphate transfection.

### Cell Culture

Human fetal RPE (hfRPE) cells were generously provided by Dr. Sheldon S. Miller, Dr. Kapil Bharti, and Dr. Arvydas Maminishkis (National Eye Institute, Bethesda, MD, United States) and cultured following the protocol published previously (Maminishkis et al., 2006). In brief, hfRPE cells were maintained in MEM medium (α modification) with N1 supplement, glutamine, non-essential amino acid, penicillin–streptomycin, taurine, hydrocortisone, triiodothyronine, and 5% fetal bovine serum (heat inactivated) at 37°C with 5% CO_2_. Human fetal RPE cells were seeded on human ECM (#354237, Corning Life Sciences, Tewksbury, MA, United States) coated 12 mm polyester (PET) Transwell^®^ inserts with 0.4 μm pores in 12-well plate (#3460, Corning Life Sciences, Tewksbury, MA, United States) with 150K cells per well. Medium was changed twice a week. At the beginning of seven weeks after seeding, hfRPE cells were infected with Lentiviral GLuc-tagged WT-Fibulin-3, GLuc-tagged R345W-Fibulin-3, or GLuc tag only at MOI 10 with 6 μg/ml hexadimethrine bromide (#H9268, MilliporeSigma, Burlington, MA, United States) for 4 h a day for 5 days, resulting in a copy number of 55 ± 9 (mean ± SEM) in WT group versus 57 ± 3 (mean ± SEM) in mutant group.

ARPE-19 Tet-On cells with Lentiviral GLuc, GLuc-tagged WT- or R345W-Fibulin-3 were described previously (Hulleman et al., 2013). Inserted genes were expressed only in the presence of Doxycycline (1 μg/ml, Dox, #D9891, MilliporeSigma, Burlington, MA, United States). ARPE-19 Tet-On cells were maintained at 37°C with 5% CO_2_ in DMEM (Dulbecco’s Modified Eagle’s Medium)/Hams F-12 50/50 Mix (#10-092-CV, Corning Life Sciences, Tewksbury, MA, United States) supplemented with 10% fetal bovine serum (FBS, #100106, BenchMark^TM^ GeminiBio, West Sacramento, CA, United States) and penicillin–streptomycin.

### Immunocytochemistry

Cells in Transwell^®^ inserts were washed twice with PBS and fixed with 4% paraformaldehyde for 15 min at room temperature. Cells were washed twice with PBS, then treated with 0.1 M glycine for 15 min and permeabilized with 0.1% Triton X-100 for three times, 2 min each. Cells were blocked with 10% normal donkey serum for 2 h at room temperature then incubated with rabbit polyclonal anti- zonula occludens-1 (ZO-1) (1:100, #61-7300, Thermo Fisher Scientific, Waltham, MA, United States) overnight at 4°C. Cells were washed three times in PBS and incubated with Alexa Fluor^®^ 488 donkey anti-rabbit IgG (H + L) for 1 h (1:500, #711-546-152, Jackson ImmunoResearch Laboratories, Inc., West Grove, PA, United States). Nuclei were counterstained with Hoechst 33342 (1 μg/ml, #B2261, MilliporeSigma, Burlington, MA, United States). The Transwell^®^ membranes with cells were mounted on microscope slides with Aqua-Poly/Mount medium (#18606-20, Polysciences, Warrington, PA, United States). Images were acquired using a Leica SP8 confocal microscope (Leica Microsystems, Wetzlar, Germany).

### Enzyme-Linked Immunosorbent Assays

Cell culture media were collected from the upper and lower chambers of Transwells after incubation for 48 h. Vascular endothelial growth factor (VEGF) enzyme-linked immunosorbent assays (ELISAs; Quantikine; R&D Systems, Minneapolis, MN, United States) were performed according to kit instructions. Optical densities were determined within 30 min with a SpectraMax 190 microplate reader (Molecular Devices, San Jose, CA, United States) at 450 nm with wavelength correction at 570 nm. All VEGF ELISA experiments were run in biological and technical triplicate.

### Luciferase Assay

Cell culture media was collected weekly from apical and basal compartments of Transwell^®^ inserts after Lentiviral infection, spun at 12,000 *g*, 15 min at 4°C, and stored at -20°C. Cells were lysed in 1× Passive Lysis Buffer (#E1941, Promega, Madison, WI, United States). *Gaussia* Luciferase activities were measured by BioLux^®^ *Gaussia* Luciferase Assay Kit (#E3300, New England BioLabs, Ipswich, MA, United States) in triplicate according to the manufacturer’s standard protocol using a Sirius Tube Luminometer (Berthold Detection Systems GmbH, Pforzheim, Germany).

### Real-Time PCR

Total RNA was extracted from samples using the AllPrep DNA/RNA/Protein Mini Kit (#80004, Qiagen Sciences, Inc., Germantown, MD, United States) according to the manufacturer’s protocol. RNA was eluted with RNase-free water. Total RNA was quantified using a NanoDrop 2000 Spectrophotometer (Thermo Fisher Scientific, Waltham, MA, United States). For reverse transcription, 0.25 μg of total RNA was used in the SuperScript^TM^ IV First-Strand cDNA Synthesis System (#18091050, Thermo Fisher Scientific, Waltham, MA, United States) with a mixture of 50 μM oligo-dT and 50 ng/μl random hexamers as primers. Real-time primers were designed with Primer3web, version 4.4.0^1^ and validated using melt curve analysis and agarose gel electrophoresis. PCR primers used in this study are shown in Supplementary Table 1. Significant gene expression was evaluated using the FastStart Universal SYBR Green Master (ROX; #4913850001, Roche Molecular Systems, Inc., Branchburg, NJ, United States) in triplicate in QuantStudio^TM^ 3 Real-Time PCR Systems (Thermo Fisher Scientific, Waltham, MA, United States). The relative quantity of mRNA was normalized to glyceraldehyde 3-phosphate dehydrogenase (GAPDH) using the comparative 2^–ΔΔCt^ method.

### Cell Migration Assay

ARPE-19 cells were cultured in 96-well ImageLock Microplates (Essen Bioscience Inc., Ann Arbor, MI, United States) to confluence (*n* = 8), and scratches were made using a 96-pin WoundMaker^TM^ (Essen Bioscience Inc., Ann Arbor, MI, United States). The wells were then washed with PBS to remove cell debris. Wound images were acquired automatically by the IncuCyte^TM^ software system (Essen Bioscience Inc., Ann Arbor, MI, United States). Images were collected at 1-h intervals for the duration of the experiment (72 h). The data were then analyzed via IncuCyte S3 Software (Essen Bioscience Inc., Ann Arbor, MI, United States) using the Relative Wound Confluence integrated metric.

### Western Blots

Western blots were performed as described previously (Zhao et al., 2018). The primary antibodies used in this study were rabbit anti-GLuc (1:5,000, #E8023, New England Biolabs, Ipswich, MA, United States) and mouse anti-GAPDH (1:1,000, #sc-32233, Santa Cruz Biotechnology, Inc., Dallas, TX, United States).

### Trans-Epithelial Electrical Resistance

Trans-epithelial electrical resistance (TER) of hfRPE cells was measured weekly after seeding in Transwell^®^ inserts using the EVOM^2^ Epithelial Voltohmmeter (World Precision Instruments, Sarasota, FL, United States). In each plate, one coated insert without cells was measured as blank and final resistance was calculated by multiplication of net resistance (R_Total_ – R_blank_, Ω) with effective membrane area (cm^2^). The electrodes were rinsed with 70% ethanol and sterile pre-warmed culture medium.

### Transmission Electron Microscopy

Cells were washed twice with PBS and fixed in 1/2 strength Karnovsky fixative (2% paraformaldehyde, 2.5% glyceraldehyde, pH 7.3) for 1 h at room temperature and further fixed in 1% osmium tetroxide in 0.1 M cacodylate buffer (pH 7.4) for 1 h. Samples were dehydrated in a graduated ethanol series terminating in pure acetone and embedded in LX-112 (Ladd Research, Williston, VT, United States). Ultrathin sections (60 nm) were stained with uranyl acetate and lead citrate and viewed with a JEOL JEM1400 Transmission Electron Microscope (JEOL USA Inc., Peabody, MA, United States), Penn State College of Medicine Imaging Core Facility.

### Statistics

Data are presented as mean ± standard error of the mean (SEM). Statistical analysis was performed using Prism (GraphPad, Inc., La Jolla, CA, United States). One-way analysis of variance and multivariate analysis were used to determine differences within groups. When identified, a student’s *t*-test was used to compare differences between groups. *p* < 0.05 was considered statistically significant.

## Results

### Primary RPE Cells Exhibit Terminal Differentiation on Transwell Filters

**FIGURE 1:**
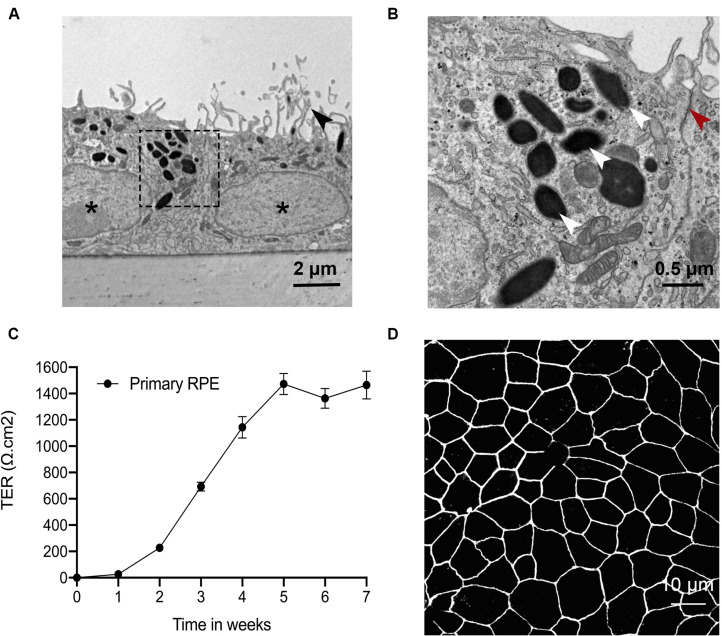
Primary RPE cells have a terminal differentiation phenotype on Transwell filters. Primary RPE cells were cultured on Transwell filters, and their phenotype was evaluated by TEM, TER and tight junction protein ICC. **(A)** RPE cells grew in a single monolayer with apical microvilli (black arrowhead), melanosomes, basal nuclei (asterisks), and membrane infoldings. **(B)** Magnification of the indicated area in A shows RPE cells with clearly visible tight junctions (red arrowhead) and melanosomes (white arrowhead). **(C)** Barrier function was assessed by quantifying TER over time. After four weeks, the TER plateaued at ∼1,400 Ω cm^2^. **(D)** ZO-1 immunoreactivity was continuous along the cell borders.

Primary RPE cells were placed on Transwell filters, and several endpoints were used to test the differentiation status at four weeks. Transmission electron microscopy (TEM) imaging showed that primary RPE cells displayed appropriate structural polarity with apically located microvilli, tight junctions, and melanosomes, and basally located nuclei and basal infoldings (Figures 1A,B). Barrier function was assessed by quantifying TER over time. After 4 weeks, the TER plateaued at ∼1,400 Ω cm^2^ (Figure 1C). Tight junction expression and distribution was confirmed by confocal imaging. Zonula occludens-1 immunoreactivity was abundant and continuous along the cell borders (Figure 1D). These data confirm that primary RPE cells grown on Transwells are polarized and differentiated.

### Expression of R345W-Fibulin-3 Activates the IRE1α/XBP1 Pathway in RPE Cells

**FIGURE 2:**
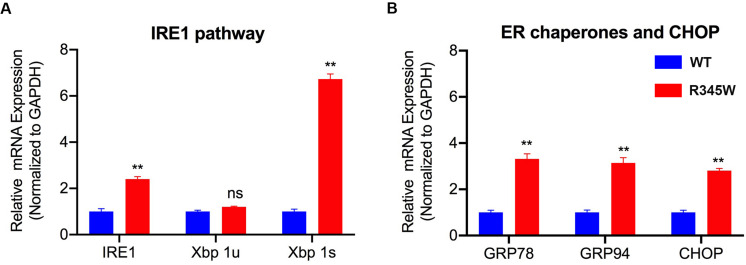
Expression of R345W-Fibulin-3 elevates markers of ER stress in primary RPE cells. mRNA isolated from primary RPE transfected with WT or R345W-Fibulin-3 was quantified by rtPCR. **(A)** IRE1α/XBP1 mRNA were elevated in primary RPE cells expressing mutant Fibulin-3 compared to controls, while Xbp 1u was unaltered. **(B)** mRNA levels of ER chaperone, GRP-78 and GRP-94, and transcription factor CHOP were also elevated compared to cells expressing WT-Fibulin-3. (*n* = 3, values are mean ± SEM, **p < 0.01).

A prior study revealed that in ARPE-19 cells, expression of R345W-Fibulin-3 activates ER stress via the IRE1α/XBP1 pathway (Roybal et al., 2005). This study showed that overexpression of R345W-Fibulin-3 induces the elevated expression of Binding immunoglobulin protein (BiP) also known as glucose regulated protein-78 (GRP-78) at both the mRNA and protein levels and induces increased expression of XBP1 at mRNA level (Roybal et al., 2005). To investigate the specific ER stress pathway(s) induced by mutant Fibulin-3 in primary RPE cells, total RNA was isolated at 14 weeks post-infection (*n* = 3), and quantified by rtPCR. Consistent with the previous study, IRE1α/XBP1 pathway and transcription factor C/EBP homologous protein (CHOP) were elevated in primary RPE cells expressing mutant Fibulin-3 compared to controls (Figure 2A). mRNA levels of ER chaperone, BiP/GRP-78 and GRP-94 were also elevated compared to cells expressing WT-Fibulin-3 (Figure 2B). These results suggest that expression of mutant Fibulin-3 activates ER stress in RPE cells primarily through the IRE1α/XBP1 pathway.

### Expression of R345W-Fibulin-3 in RPE Cells Disrupts Tight Junction Protein Distribution and Impairs Barrier Function

**FIGURE 3:**
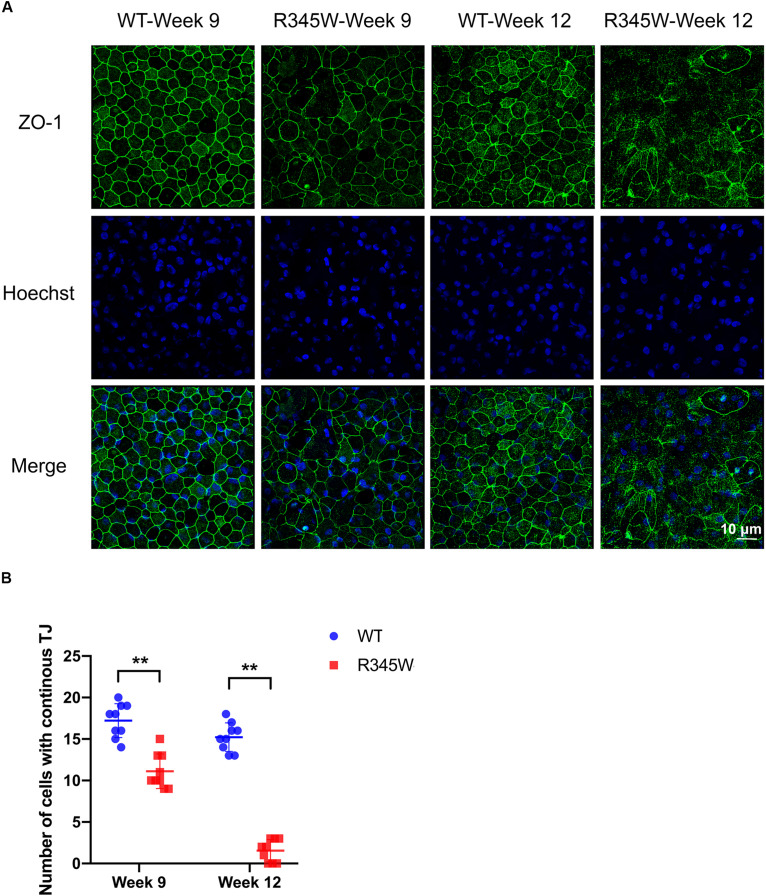
ZO-1 distribution is disrupted in primary RPE cells expressing R345W-Fibulin-3. The expression and distribution of ZO-1 tight junction protein was assessed in primary RPE cells 9 and 12 weeks after infection with WT- or R345W-Fibulin-3 **(A)** Maximum projection confocal images of ZO-1 ICC. Green: ZO-1; Blue: Hoechst-stained nuclei. Confocal imaging reveals that ZO-1 distribution was dramatically disrupted in RPE cells expressing R345W-Fibulin-3 at 9 and 12 weeks post-infection. **(B)** Cells with continuous ZO-1 distribution were counted in nine different areas of 625 μm^2^. The number of cells with continuous tight junctions was significantly lower in RPE cells expressing R345W-Fibulin-3 at 9 and 12 weeks post-infection compared to WT infected controls (*n* = 9, values are mean ± SEM, **p < 0.01).

To investigate the effects of R345W-Fibulin-3 expression on RPE permeability barrier function, we first examined ZO-1 distribution using the primary RPE cell culture system described above. Immunocytochemistry (ICC) was conducted at 9 or 12 weeks post-infection. Confocal imaging revealed that ZO-1 distribution was dramatically disrupted and disorganized in the mutant group relative to the WT group (Figure 3A). To quantify these changes, the number of RPE nuclei associated with continuous ZO-1 was counted in nine regions of the 625 μm^2^ confocal image. Relative to controls, continuous ZO-1 distribution in the mutant group was reduced by 38% (*p* < 0.01) after 9 weeks of infection and by 90% after 12 weeks (*p* < 0.01; Figure 3B). These data indicate that the expression of mutant Fibulin-3 in RPE cells disrupts tight junction protein distribution.

**FIGURE 4:**
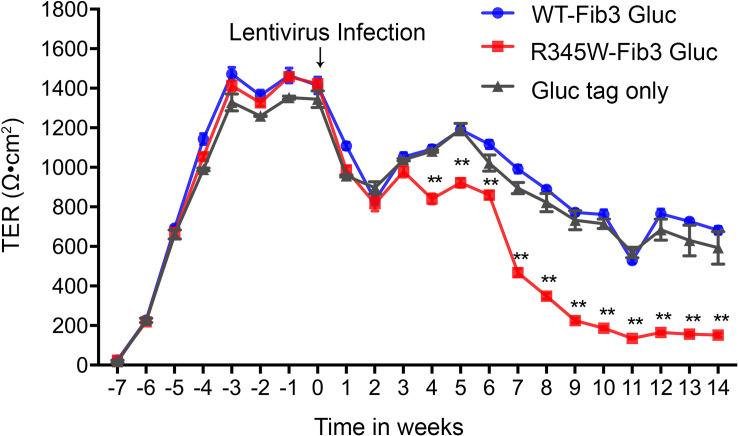
Expression of R345W-Fibulin-3 decreases TER in primary RPE cells. Cell permeability was assessed by monitoring TER in RPE cells once a week for 21 weeks. At the beginning of the 4th week (week-3 on *x*-axis), RPE cells reached maturity with TER values maximizing between 1,300 and 1,500 Ω cm^2^. In the beginning of the 7th week (week 0 on *x*-axis), RPE cells were infected with lentivirus with either luciferase-tagged WT-Fibulin-3 (blue), luciferase-tagged R345W-Fibulin-3 (red), or luciferase-tag only (gray) for five consecutive days. At 3 weeks post-infection, TER decreased to approximately 800 Ω cm^2^ in all groups. At 4 weeks post-infection, TER continuously dropped in the mutant group (*n* = 8) and reached values significantly lower than the WT (*n* = 8) and tag-only groups (*n* = 2). (Values are mean ± SEM of individual wells, **p < 0.01).

To assess the impact of disrupted tight junction protein distribution on a critical aspect of RPE cell function, TER was monitored over time. Four weeks after being seeded, the TER of all cells reached a plateau of approximately 1,400 Ω cm^2^. Seven weeks after seeding, cells were infected with GLuc-tagged WT-Fibulin-3, GLuc-tagged R345W-Fibulin-3, or GLuc tag only. At 3 weeks post-infection, the TER was reduced to 800–1,000 Ω cm^2^ in all three groups, but no significant differences were observed between groups. Starting at 4 weeks post-infection, the TER of cells expressing R345W-Fibulin-3 was significantly reduced relative to the GLuc-tagged WT-Fibulin-3 and GLuc tag only groups. Trans-epithelial electrical resistance in the GLuc-tagged R345W-Fib3 group was 70.7% lower at 9 weeks post-infection (*n* = 8, *p* < 0.01) and 78.4% lower at 12 weeks post-infection (*n* = 8, *p* < 0.01; Figure 4). These data are consistent with the altered ZO-1 distribution described above and further indicate that expression of mutant Fibulin-3 induces loss of RPE barrier function.

### Expression of R345W-Fibulin-3 in Primary RPE Cells Reduces the Basal Secretion of VEGF

**FIGURE 5:**
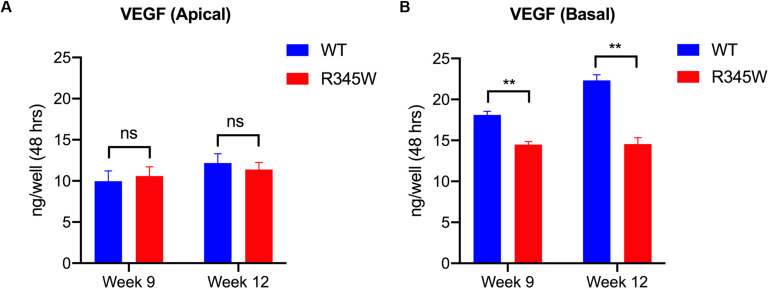
VEGF secretion is impaired in primary RPE cells expressing R345W-Fibulin-3. VEGF secretion levels were quantified by ELISA. Cell culture media were collected from the apical and basolateral sides of primary RPE cells on Transwell filters at 9 and 12 weeks post-infection. **(A)** Apical VEGF secretion was not significantly different in the R345W-Fibulin-3 cells compared to WT, **(B)** Basal VEGF secretion was significantly lower in the mutant group compared to the WT group at 9 and 12 weeks post-infection. Values are the mean ± SEM of individual wells (*n* = 8, **p < 0.01).

It is well established that polarized secretion of proteins by RPE cells is critical for RPE, photoreceptor, and choroidal homeostasis, and that the polarized secretion of VEGF is of particular significance (Bhutto et al., 2006; Maminishkis et al., 2006). In terminally differentiated RPE cells, VEGF is primarily secreted in the basal direction to promote the growth of the choroidal vasculature (Marneros et al., 2005). To test whether mutant Fibulin-3 induction alters the polar secretion of growth factors, we quantified apical and basal VEGF secretion using the primary RPE cell culture system described above. Cell culture medium was collected from the apical and basal compartments of each well. VEGF165 was quantified using a validated ELISA. The apical VEGF concentration did not differ between groups (Figure 5A). Compared to WT-Fibulin-3, basal VEGF secretion was significantly lower in the R345W-Fibulin-3 group at both 9 and 12 weeks post-infection (*n* = 8, *p* < 0.01; Figure 5B). These results suggest that expression of mutant Fibulin-3 alters the polarized secretion of VEGF.

### Expression of R345W-Fibulin-3 Alters Its Polarized Secretion in Primary RPE Cells

**FIGURE 6:**
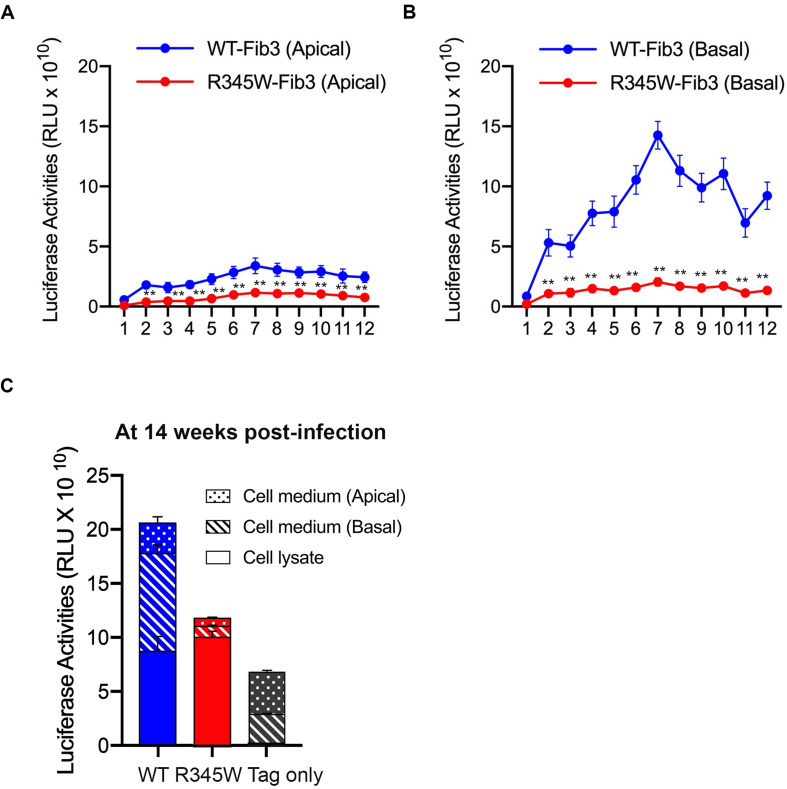
Secretion of R345W-Fibulin-3 is impaired in both apical and basolateral directions. Fibulin-3 secretion levels were monitored by luciferase assay from media collected from the apical and basolateral sides of primary RPE cells. **(A,B)** Fibulin-3 was preferentially secreted to the basal side in both WT and mutant cells. R345W-Fibulin-3 secretion levels in mutant cells were significantly lower than those of WT cells over time (*n* = 8, **p < 0.01). **(C)** After 14 weeks of infection, primary RPE cells were harvested and cell media were collected. No significant differences in luciferase activities between the WT and mutant groups were observed. Luciferase activities were decreased in RPE cells expressing R345W-Fibulin-3 in both the apical and basolateral directions.

To monitor the secretion of WT-Fibulin-3 and R345W-Fibulin-3, cell culture media were collected once a week for 12 weeks following infection. The results revealed that luciferase activity in basal cell culture media was significantly higher than that of apical cell culture media from RPE cells expressing both WT and mutant forms of Fibulin-3 (*n* = 8, *p* < 0.01; Figure 6A). Luciferase activity in the cell culture media of primary RPE cells expressing mutant Fibulin-3 was significantly lower than that of cells expressing the WT form on both apical and basal sides. Moreover, we found that R345W-Fibulin-3 secretion was severely impaired in the basal direction and moderately impaired apically. The decreased basal:apical secretion ratio of R345W-Fibulin-3 suggests that R345W-Fibulin-3 expression impairs polarized Fibulin-3 secretion in RPE cells (*n* = 8, *p* < 0.01; Figures 6A,B).

At 14 weeks post-infection, cell lysates and cell culture media were harvested, and luciferase activity was quantified via a luciferase assay. Our results showed no significant differences in primary RPE cell lysate luciferase activities between WT and mutant groups, which is not consistent with previous studies in RPE-J cells or ARPE-19 cells that reported greater amounts of mutant Fibulin-3 within cells relative to the WT protein (Marmorstein et al., 2002; Roybal et al., 2005). One possible explanation for the differing results is the differences between these cell culture systems. Consistent with previous studies (Hulleman et al., 2011), luciferase activity in the cell culture media of RPE cells expressing mutant Fibulin-3 was 73.6 and 88.8% lower on the apical and basal sides, respectively, than that of cells expressing the WT form after 14 weeks of infection (*n* = 8, *p* < 0.01; Figure 6C). The reduction in total luciferase activity including both cell lysates and cell culture media in the mutant group may due to the increased degradation of mutant Fibulin-3 and/or RPE dysfunction at later time points.

### Expression of R345W-Fibulin-3 Leads to Downregulated Expression of RPE Signature Genes and Upregulated Expression of EMT Markers

**FIGURE 7:**
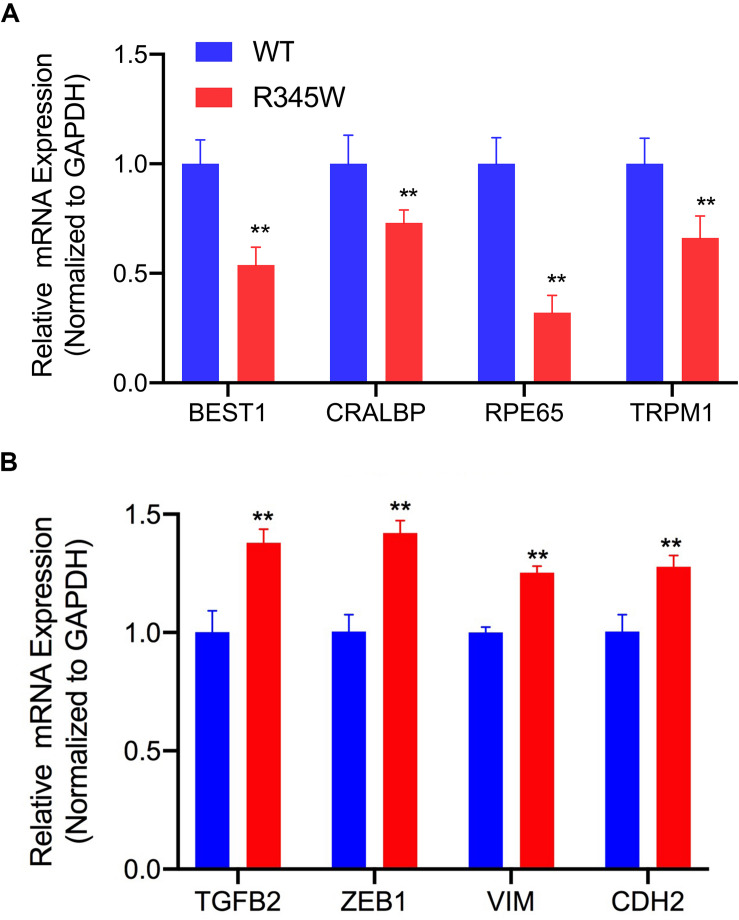
RPE signature genes are downregulated and EMT markers are upregulated in primary RPE cells expressing R345W-Fibulin-3. **(A)** At 14 weeks post-infection, primary RPE cells were harvested and total RNA was isolated. RT-PCR was performed to quantify four RPE signature genes (BEST1, CRALBP, RPE65, and TRPM1). All expression levels are normalized to GAPDH expression. Relative to the WT group, mRNA for BEST1, CRALBP, RPE65, and TRPM1 were significantly reduced in RPE cells expressing R345W-Fibulin-3 (*n* = 3, **p < 0.01). **(B)** At 14 weeks post-infection, primary RPE cells were harvested, and total RNA was isolated. RT-PCR was performed to quantify four EMT markers, (TGFB2, ZEB1, VIM, and CDH2). All expression levels were normalized to GAPDH expression. The experimental results showed that, relative to the WT group, TGFB2, ZEB1, VIM, and CDH2 were significantly upregulated in RPE cells expressing R345W-Fibulin-3 (*n* = 3, **p < 0.01).

To determine the differentiation state of the RPE cells in culture, we evaluated the expression levels of validated RPE cell signature genes. These genes were defined as mean expression values upregulated 10-fold or greater in native adult RPE cells relative to other cell types (Liao et al., 2010; Strunnikova et al., 2010). BEST1 (bestrophin-1), CRALBP (retinaldehyde-binding protein), RPE65 (retinal pigment epithelium-specific 65 kDa protein), and TRPM1 (transient receptor potential cation channel) are four RPE cell signature genes with functions in membrane transport, the visual cycle, and pigmentation pathways. We investigated whether R345W-Fibulin-3 expression influences RPE signature gene expression using the same cell culture system as above. After 14 weeks of infection, primary RPE cells were harvested for total RNA isolation. RT-PCR was conducted to examine signature gene expression in each group. BEST1, CRALBP, RPE65, and TRPM1 expression levels were normalized to GAPDH expression. RT-PCR results showed that BEST1, CRALBP, RPE65, and TRPM1 expression levels were significantly lower in the GLuc-tagged R345W-Fibulin3 group than in the GLuc-tagged WT-Fibulin-3 group (*n* = 3, *p* < 0.01), indicating that the expression of R345W-Fibulin-3 attenuates RPE cell differentiation (Figure 7A).

It is thought that cell dedifferentiation is usually followed by trans-differentiation, namely, EMT, due to stress-induced cellular reprogramming which includes increased cell migration (Pallet, 2012; Johno and Kitamura, 2013; Lamouille et al., 2014). Using RT-PCR, we compared the expression levels of TGFB2 (transforming growth factor beta-2), ZEB1 (Zinc Finger E-Box Binding Homeobox 1), VIM (Vimentin), and CDH2 (Cadherin 2) across groups using the primary RPE cell culture system described above. Compared to the WT group, the expression levels of all four EMT markers were significantly higher in the mutant group (*n* = 3, *p* < 0.01). Notably, the magnitude of EMT induction was similar to prior work (Wang et al., 2010; Adijanto et al., 2012), suggesting that expression of R345W-Fibulin-3 facilitates EMT in RPE cells (Figure 7B).

### Expression of R345W-Fibulin-3 Leads to Enhanced Migration Ability in RPE Cells

To evaluate whether the expression of R345W-Fibulin-3 leads to migratory changes in RPE cells, scratch assays were conducted in ARPE-19 cells overexpressing either a dox-inducible GLuc-tagged WT-Fibulin-3 or R345W-Fibulin-3. We examined WT-Fibulin-3 and R345W-Fibulin-3 expression in this Tet-On ARPE-19 cell system by western blot. We found that the non-induced mutant group displayed a small but detectable amount of leaky expression of R345W-Fibulin-3 (Supplementary Figure 1). In the cell culture media, the amount of “leaky” expression in the absence of dox is 7% (GLuc-tagged WT) and 34% (GLuc-tagged R345W) of the respective western blot signal with dox treatment for 48 h. In the cell lysate, the amount of “leaky” expression in the absence of dox is 3% (GLuc-tagged WT) and 3% (GLuc-tagged R345W) of the respective western blot signal with dox treatment for 48 hours, consistent with previous study (Hulleman et al., 2013).

**FIGURE 8:**
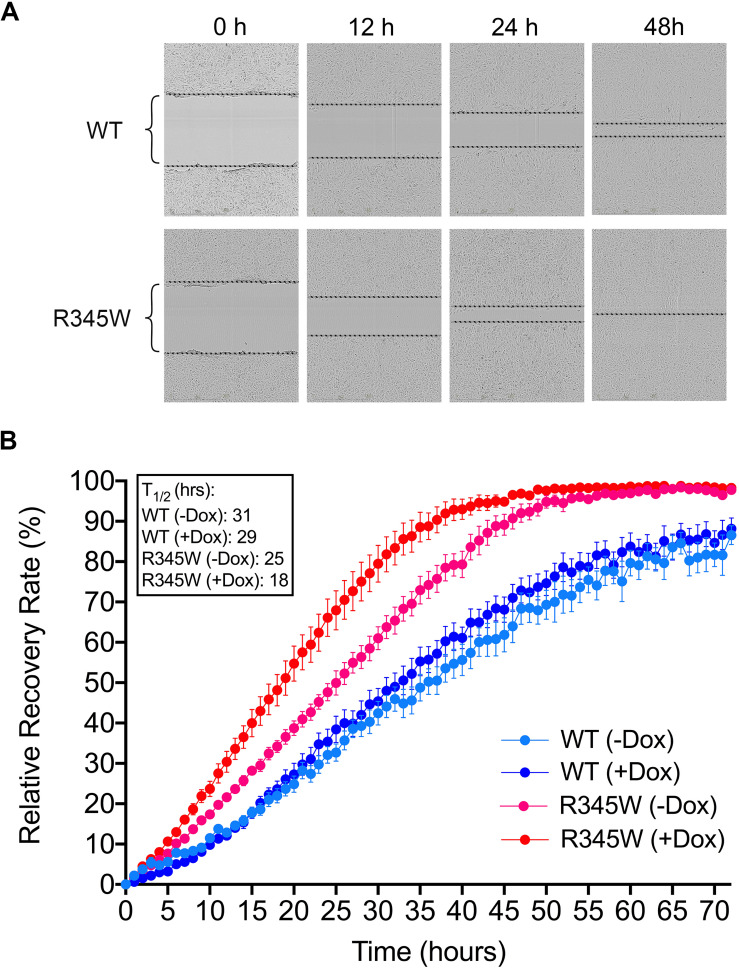
Expressing R345W-Fibulin-3 increases cell migration. Scratch assays were conducted in ARPE-19 cells overexpressing either WT-Fibulin-3 or R345W-Fibulin-3. **(A,B)** The experimental results showed that, relative to the WT group, ARPE-19 cells overexpressing R345W-Fibulin-3 had a significantly faster scratch recovery rate in would healing assays. Brackets represent wound distances (*n* = 8, *p* < 0.01).

Expression of R345W-Fibulin-3 enhanced migration ability in a dose-dependent manner. Scratches were made after 48 h of dox-induced expression of either WT-Fibulin-3 or R345W-Fibulin-3 in ARPE-19 cells. Relative wound confluence was calculated automatically by the IncuCyte^TM^ software system at one-hour intervals for 72 h (Figure 8). Scratch recovery rate was calculated as time to 50% wound closing (T_1__/__2_). We found that dox-induced mutant group had the fastest time for closing 50% of the wound (T_1__/__2_ = 18 h), suggesting that the expression of R345W-Fibulin-3 increased the migration ability of RPE cells. Compared to the non-induced WT (T_1__/__2_ = 31 h) and dox-induced WT groups (T_1__/__2_ = 29 h), the non-induced mutant group had a faster scratch recovery rate (T_1__/__2_ = 25 h), and, relative to the dox-induced mutant group, it had a slower scratch recovery rate (*n* = 8, *p* < 0.01; Figures 8A,B).

## Discussion

The current study sheds light on the potential impact of misfolded protein accumulation due to the R345W mutation in fibulin-3 in RPE cells. In this study, we cultured primary RPE cells on Transwells and observed their morphology under TEM. As in studies presented by others, our primary RPE cells showed robust tight junctions and structural polarity under TEM (Maminishkis et al., 2006; Sonoda et al., 2009; Maminishkis and Miller, 2010). Unfolded protein response activation involves several mechanisms aimed at reducing the load of aberrant protein accumulation, including attenuated protein translation to avoid worsening the accumulation, increased transcription of ER chaperones, including GRP-78 and GRP-94 to aid in the folding process, and an increase in ER-associated degradation (ERAD). Three ER transmembrane sensors, IRE1, PERK, and ATF6, regulate the UPR and determine the appropriate adaptive response, directing the cell to proliferate, change shape, or undergo apoptosis. Prior studies have also shown that mutant Fibulin-3 is misfolded and accumulated in the ER (Marmorstein et al., 2002; Roybal et al., 2005). Our study showed that expression of mutant Fibulin-3 induces increased expression of ER chaperones and IRE1/XBP1 pathway. We found that, in primary RPE cells expressing R345W-Fibulin-3, barrier function was impaired, as evidenced by disrupted ZO-1 distribution and decreased TER. This is in line with a prior study which showed that RPE cells retained a differentiated phenotype if they maintained cell-cell contacts, whereas EMT was initiated when RPE cells lost tight junctions, suggesting that cell-cell contact plays an important role in the RPE transition from a well-differentiated phenotype to a fibroblast phenotype. This phenotypic switch may contribute to the development of fibrotic complications (Tamiya et al., 2010).

We next examined the effects of R345W-Fibulin-3 expression on the polarized secretion of proteins by RPE cells. In fully polarized RPE cells, VEGF is preferentially secreted to the basal side of the RPE monolayer for choroidal vasculogenesis, a function that may be impaired in AMD. For example, decreased thickness of the choroidal layer has been observed in AMD patients (Lee et al., 2013), while a previous study showed that RPE de-differentiation causes atrophy of the choriocapillaris (Ohlmann et al., 2016). Our data are consistent with these findings, whereby the levels of basally secreted VEGF were significantly lower in the mutant group, indicating that the polarized secretion of VEGF was disrupted in RPE cells expressing R345W-Fibulin-3.

To our knowledge, this is the first study to delineate the directionality of impaired R345W-Fibulin-3 secretion. We found that WT-Fibulin-3 is preferentially secreted in the basal direction, substantiating the hypothesis that Fibulin-3 plays a role in maintaining the RPE cell basement membrane (Giltay et al., 1999; Segade, 2010). We further found that R345W-Fibulin-3 secretion is severely impaired on the basal side and moderately impaired in the apical direction. These results suggest that expression of R345W-Fibulin-3 impairs polarized Fibulin-3 secretion in RPE cells. The reduction in total secretion of R345W Fibulin-3 may be due to accelerated degradation or to excessive intracellular accumulation.

**FIGURE 9:**
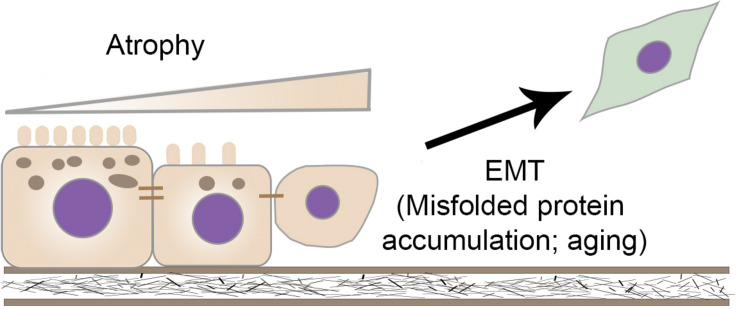
Working model for RPE cells dysfunction and undergoing EMT. RPE cells exhibit a well-differentiated epithelial phenotype, characterized by integrated tight junctions, a high degree of polarity, and proper RPE signature gene expression. Expression of R345W-Fibulin-3 in RPE cells leads to a mesenchymal-like phenotype with cytoskeletal changes, a transcriptional shift, and an increased capacity for migration. Potential mechanisms contributing to EMT in RPE cells include aging and misfolded protein accumulation.

We next showed that RPE cell signature genes were downregulated in primary RPE cells expressing R345W-Fibulin-3. Moreover, our data showed that four EMT markers, TGFB2, ZEB1, VIM, and CDH2, were upregulated and migration ability was enhanced in the mutant group, suggesting that the expression of R345W-Fibulin-3 not only attenuates RPE cell differentiation, but also facilitates EMT of RPE cells. This may explain the formation of sub-RPE deposits and increased thickness of Bruch’s membrane in macular degeneration, as more extracellular matrix proteins are secreted by mesenchymal-like cells than epithelial cells. In addition, increased migration ability in mutant RPE cells may explain the presence of HRF in OCT images, which have been shown to display characteristics of RPE migration in published studies. Figure 9 illustrates our working model for RPE cell dedifferentiation and EMT.

The current study shows that Fibulin-3 mutation leads to a phenotypic shift in RPE cells. However, the specific mechanisms by which mutant Fibulin-3 leads to EMT of RPE cells are unclear. Previous studies have shown that expression of mutant Fibulin-3 causes activation of the UPR in ARPE-19 cells (Roybal et al., 2005). Emerging evidence suggests that the accumulation of misfolded proteins drives EMT via activation of the UPR (Mo et al., 2015; Cuevas et al., 2017; Santamaria et al., 2019). Thus, UPR activation may constitute one of the underlying mechanisms by which RPE cells undergo EMT.

In summary, our experimental results have shown that native and WT-fibulin-3 overexpressing RPE cells exhibit a terminally differentiated epithelial phenotype with continuous, barrier-forming tight junctions, high polarization, and high expression of RPE signature genes. The expression of R345W-Fibulin-3 causes RPE cells to undergo EMT, as evidenced by upregulated EMT markers and an increased migration ability. The findings from this study will help us gain a better understanding of the role of misfolded proteins in RPE dysfunction.

## Data Availability Statement

The original contributions presented in the study are included in the article/Supplementary Material, further inquiries can be directed to the corresponding author/s.

## Author Contributions

MZ, YZ, and JS conceived and planned the experiments. MZ, YZ, and HC carried out the experiments. MZ, SW, YZ, AB, SG, CW, HW, JH, and JS contributed to sample preparation and interpretation of the results. MZ, SW, and JS took the lead in writing the manuscript. JS conceived the original idea and supervised the project. All authors provided critical feedback and helped shape the research, analysis and manuscript.

## Conflict of Interest

The authors declare that the research was conducted in the absence of any commercial or financial relationships that could be construed as a potential conflict of interest.

## Footnotes

1 http://bioinfo.ut.ee/primer3/

## Supplementary Material

FIGURE S1 Western blot analysis of Fibulin-3 secretion and expression levels in ARPE-19 cells. Secretion and expression levels of WT-Fibulin-3 and R345W-Fibulin-3 were quantified in the cell culture media and cell lysate, respectively, from the Tet-On ARPE-19 cells by western blot. The results showed that, relative to R345W-Fibulin-3, more WT-Fibulin-3 was secreted into the cell culture media. Relative to WT-Fibulin-3, more R345W-Fibulin-3 was retained within the cells. Without dox induction, GLuc-tagged WT-Fibulin-3 and R345W-Fibulin-3 were still weakly detected in both cell culture media and cell lysates, suggesting slightly leaky Fibulin-3 expression in this Tet-On ARPE-19 cell system.

TABLE S1 PCR primers used in this paper.
