# Supplementary figures and images for: Expression of R345W-Fibulin-3 Induces Epithelial-Mesenchymal Transition in Retinal Pigment Epithelial Cells
Source: Front Cell Dev Biol. 2020 Jun 19;8:469. doi: 10.3389/fcell.2020.00469 (PMC7317295; doi:10.3389/fcell.2020.00469)

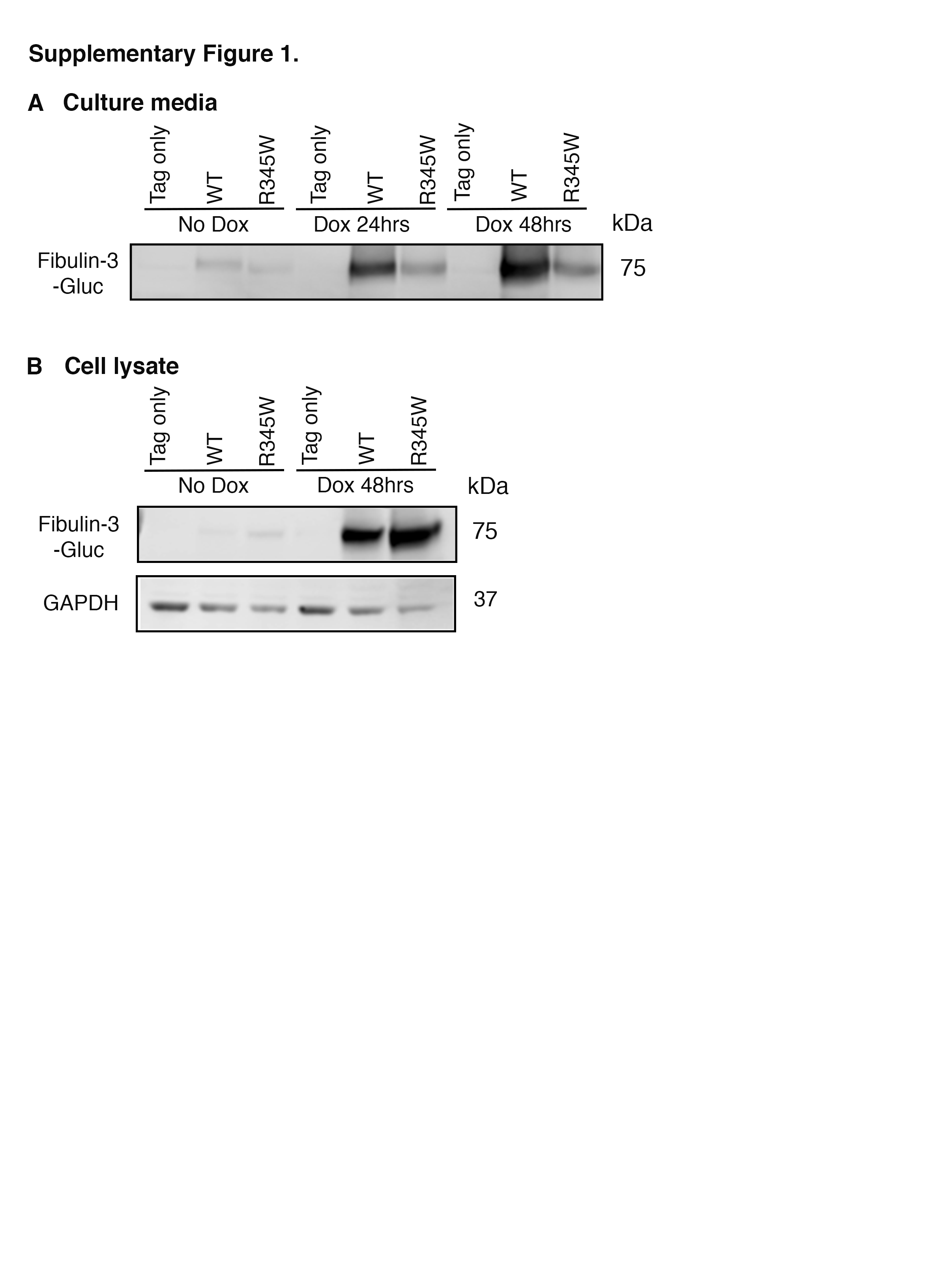

Supplement: FIGURE S1 — Western blot analysis of Fibulin-3 secretion and expression levels in ARPE-19 cells. Secretion and expression levels of WT-Fibulin-3 and R345W-Fibulin-3 were quantified in the cell culture media and cell lysate, respectively, from the Tet-On ARPE-19 cells by western blot. The results showed that, relative to R345W-Fibulin-3, more WT-Fibulin-3 was secreted into the cell culture media. Relative to WT-Fibulin-3, more R345W-Fibulin-3 was retained within the cells. Without dox induction, GLuc-tagged WT-Fibulin-3 and R345W-Fibulin-3 were still weakly detected in both cell culture media and cell lysates, suggesting slightly leaky Fibulin-3 expression in this Tet-On ARPE-19 cell system. [file Image_1.jpeg]
